# Efficacy of alpha-blockers in medical expulsive therapy for ureteral stones: A systematic review and meta-analysis of randomized controlled trials between 2010 and 2025

**DOI:** 10.1080/20905998.2025.2532196

**Published:** 2025-07-29

**Authors:** Mohammed Marzouq Almaghthawi, Eman Abdullah Alotaibi, Mohammed Saad Alotaibi, Renad Wesam Alomari, Yazeed Dakhel Alsulami, Manar Ali Alahamdi, Salem Ibrahim S Aljaddua, Wijdan Ateeq Allah Alruhaili, Eyad Mohammed Hijazi, Abdullah Sulaiman Alkharboosh, Oroub Abdulaziz Almurshed, Adel H. Alshammari

**Affiliations:** a College of Medicine, Qassim University, Unayzah city, Al-Mulaida, Saudi Arabia; b Department of Family and Community Medicine, College of Medicine, Qassim University, Unayzah city, Al-Mulaida, Saudi Arabia; c College of Pharmacy, Umm Al-Qura University, Makkah Al-Mukarramah, Saudi Arabia; d College of Medicine, King Saud bin Abdulaziz University for Health Sciences, Jeddah, Saudi Arabia; e College of Pharmacy, Taibah University, Al Madinah Al Munawwarah, Saudi Arabia; f College of Medicine, Jouf University, Al-Jouf, Saudi Arabia; g College of Medicine, Taibah University, Al Madinah Al Munawwarah, Saudi Arabia; h College of Medicine, Alfaisal University, Riyadh, Saudi Arabia; i College of Medicine, King Saud bin Abdulaziz University for Health Sciences, Riyadh, Saudi Arabia; j College of Medicine, Qassim University, Al-Qassim, Saudi Arabia; k General Practice, Hafar albatin Cluster, MCH, Hafar Alnatin, Saudi Arabia

**Keywords:** Alpha-blockers, ureteral stones, urolithiasis, medical expulsive therapy, terazosin

## Abstract

**Introduction:**

Alpha-blockers are widely used in medical expulsive therapy (MET) for ureteral stones; however, the current evidence regarding their comparative effectiveness remains inconsistent. We aimed to evaluate the efficacy and safety of different alpha-blockers in facilitating ureteral stone passage and identify factors influencing treatment outcomes.

**Methods:**

We conducted a systematic review and meta-analysis of randomized controlled trials (RCTs) published between 2010 and 2025. We searched multiple databases for studies comparing alpha-blockers with control interventions or other alpha-blockers for ureteral stones ≤10 mm. Primary outcome was stone expulsion rate; secondary outcomes included time to expulsion, pain episodes, analgesic use, and adverse events. We performed subgroup analyses by alpha-blocker type, stone size, location, and treatment duration. Network meta-analysis assessed comparative effectiveness between agents.

**Results:**

Twenty-nine RCTs with a total of 4,256 patients were included. Alpha-blockers significantly increased stone expulsion rates compared to controls (70.9% vs. 56.5%; RR 1.25, 95% CI 1.20–1.32; Number Needed to Treat (NNT) = 7) and reduced expulsion time by approximately three-days. Efficacy was greatest for distal ureteral stones (RR 1.52; Number Needed to Treat (NNT) = 4) and stones 5–10 mm (RR 1.35; NNT = 6). Network meta-analysis revealed efficacy ranking favoring at first terazosin, followed by doxazosin then, silodosin then, tamsulosin then, alfuzosin and last the least effective was naftopidil. Alpha-blockers significantly reduced pain episodes and analgesic requirements. Adverse events were infrequent (Number Needed to Harm (NNH) = 38), with retrograde ejaculation being most common with silodosin.

**Conclusion:**

Alpha-blockers significantly improve the stone expulsion rates and reduce expulsion time, especially for distal ureteral stones 5–10 mm in size. While tamsulosin remains the most studied agent, our network meta-analysis suggests terazosin and doxazosin may offer superior efficacy. The favorable risk-benefit profile supports routine use of alpha-blockers for appropriately selected patients with ureteral stones.

## Introduction

Urolithiasis represents one of the most common urological disorders from all over the world, affecting around 10% of the global population with increasing prevalence rates across developed and developing countries alike [1]. We recognize that ureteral stones especially account for a significant proportion of the total population of urolithiasis cases and present significant challenges in their management. These stones, especially when lodged in the ureter, can cause excruciating pain, urinary obstruction, and may lead to consequent complications including infection, hydronephrosis, as well as renal impairment if left untreated in the correct manner timely [2].

We observe that the management of ureteral stones has advanced considerably over recent decades. While watchful waiting remains appropriate for small stones with high likelihood of spontaneous passage, more invasive approaches such as extracorporeal shock wave lithotripsy (ESWL), ureteroscopy, and percutaneous nephrolithotomy are often indicated for larger stones or those failing conservative management [3]. However, we acknowledge that these interventional procedures carry multiple risks including bleeding, infection, and ureteral injury, alongside the associated healthcare costs and resource utilization. In this manner, we recognize the importance of medical expulsive therapy (MET) as a noninvasive approach to facilitate stone passage and may reduce the need for surgical intervention as a first-line management in the eligible patients [4].

We understand that among various pharmacological agents investigated for MET, alpha-adrenergic receptor antagonists (alpha-blockers) have emerged as promising treatment line [5]. We appreciate that the scientific rationale for their use centers on the abundant distribution of alpha-1 adrenergic receptors, mostly the alpha-1D subtype, throughout the ureteral smooth muscle [5]. By antagonizing these receptors, we recognize that alpha-blockers can decrease intraureteral pressure proximal to the stone, reduce peristaltic amplitude and frequency, and increase fluid transport, thereby play a possible role in facilitating stone passage and alleviating pain [5].

Past studies have studied several alpha-blockers, including tamsulosin, silodosin, alfuzosin, doxazosin, terazosin, and naftopidil, for MET with varying specificity for receptor subtypes. Tamsulosin, a selective alpha-1A/alpha-1D antagonist, has been the most extensively studied and is widely considered a first-line therapy [6]. However, we acknowledge that more recent studies suggest that highly selective agents like silodosin (alpha-1A selective) or naftopidil (alpha-1D predominant) may offer superior efficacy or improved side effect profiles for specific patient populations [7]. Despite the growing number of clinical trials, we recognize that direct comparative evidence between different alpha-blockers remains limited, and their relative efficacy and safety profiles require further investigation [8].

Previous meta-analyses have resulted with various results regarding the effectiveness of alpha-blockers in MET, with some supporting their routine use while others questioning their benefit beyond certain subgroups [9,10]. In addition to that, we observed that most of the existing reviews have not focused to compare the entire spectrum of available alpha-blockers or adequately addressed important considerations about the agent selection based on stone characteristics, patient demographics, and healthcare settings [11].

In light of these gaps, we aim to conduct a systematic review and meta-analysis of randomized controlled trials (RCTs) to assess the efficacy and safety of alpha-blockers in medical expulsive therapy for ureteral stones in a more exposed and precise manner. We are going to evaluate stone expulsion rates, expulsion time, pain control, and adverse events across different alpha-blocker agents. We seek to determine the comparative effectiveness of various alpha-blockers through network meta-analysis, providing relevant rankings of these agents. Furthermore, we look to investigate the impact of stone size, location, and treatment duration on therapeutic outcomes.

## Methods

### Protocol and registration

Our study was conducted in accordance with the Preferred Reporting Items for Systematic Reviews and Meta-Analyses (PRISMA) 2020 guidelines [12]. We prospectively registered our study protocol with PROSPERO (International Prospective Register of Systematic Reviews; registration ID: CRD42025642470).

### Eligibility criteria

We used the PICOTS (Population, Intervention, Comparison, Outcome, Timing, Setting) framework to define our eligibility criteria. Studies were included if they met the following criteria: (1) adult patients (≥18 years) with symptomatic ureteral stones ≤10 mm confirmed by imaging (CT, ultrasonography); (2) intervention involving alpha-blockers (tamsulosin, silodosin, alfuzosin, doxazosin, terazosin, or naftopidil) as standalone or adjunctive treatment for MET; (3) comparison with placebo, standard therapy, or alternative pharmacological interventions; (4) reporting of at least one primary outcome (stone expulsion rate, time to stone expulsion, or adverse events); (5) RCT design; and (6) publication between January 2010 and January 2025 in English language.

We excluded studies involving patients with stones >10 mm, multiple stones, non-ureteral stones, bilateral stones, or associated anatomical/genitourinary abnormalities; non-alpha-blocker interventions without control or placebo comparison; studies lacking clear reporting of primary or secondary outcomes; and non-RCTs, retrospective studies, or observational studies.

### Information sources and search strategy

We conducted a databases-based literature search in January 2025 across, PubMed, Google Scholar, OVID, and Web of Science. Our search strategy has utilized the following keywords and their combinations: ‘ureteral stones,’ ‘ureterolithiasis,’ ‘ureteral calculi,’ ‘alpha-blockers,’ ‘tamsulosin,’ ‘silodosin,’ ‘alfuzosin,’ and ‘medical expulsive therapy.’ We adapted search terms for each database while maintaining consistency in search concepts.

### Study selection process

Reviewers have screened titles and abstracts of all identified citations using Rayyan systematic review management platform (https://www.rayyan.ai/). Full texts of preliminary eligible studies were then retrieved and independently assessed according to our predefined inclusion criteria. We documented the entire selection process in a PRISMA flow diagram, recording the reasons for exclusion at the full-text screening stage.

### Data extraction

We extracted the following information from each included study: (1) study characteristics (first author, publication year, country, study design); (2) participant demographics and baseline characteristics (age, sex, stone size, stone location); (3) intervention details (type and dosage of alpha-blockers, duration of treatment); (4) comparison group characteristics; (5) outcomes (stone expulsion rate, time to stone expulsion, pain episodes, analgesic use, need for intervention, adverse events) and (6) follow-up duration.

### Risk of bias assessment

We assessed the methodological quality of included studies using second version of the Cochrane Risk of Bias (RoB 2) tool for RCTs. This tool evaluates bias across five domains: (1) randomization process, (2) deviations from intended interventions, (3) missing outcome data, (4) measurement of the outcome, and (5) selection of reported results. Two researchers independently assessed each study, with disagreements resolved by a third reviewer. Each domain was classified as ‘low risk,’ ‘some concerns,’ or ‘high risk.’ The overall risk of bias for each study was determined according to Cochrane guidelines, with studies classified as ‘low risk,’ ‘some concerns,’ or ‘high risk’ based on domain-specific judgments.

### Effect measures and synthesis methods

For dichotomous outcomes (as stone expulsion rate, adverse events), we calculated risk ratios (RR) with 95% confidence intervals (CI). For continuous outcomes (as time to stone expulsion, pain episodes), we calculated mean differences (MD) or standardized mean differences (SMD) with 95% CI when different scales were used. We performed meta-analyses using RStudio software with R version 4.4.2. Based on the heterogeneity between studies, we utilized random-effects models using the DerSimonian and Laird method for our analyses. For key outcomes, we calculated the number needed to treat (NNT) for beneficial outcomes and the number needed to harm (NNH) for adverse events. We also calculated clinical significance thresholds based on patient-important differences reported in the literature.

### Assessment of heterogeneity

We assessed the statistical heterogeneity using the Cochran’s Q test (with P-value <0.10 indicating statistically significant heterogeneity) and the I^2^ statistic. I^2^ values of 25%, 50%, and 75% were interpreted as low, moderate, and high heterogeneity, respectively. For outcomes with high heterogeneity (I^2^ > 50%), we conducted subgroup analyses and sensitivity analyses to explore the possible contributing sources of heterogeneity.

### Subgroup and sensitivity analyses

We performed the subgroup analyses based on: (1) type of alpha-blocker (tamsulosin, silodosin, alfuzosin, doxazosin, terazosin, naftopidil); (2) stone size (< 5 mm vs. 5–10 mm); (3) stone location (proximal, mid, distal ureter); and (4) treatment duration (< two-weeks, 2–4 weeks, > four-weeks). Sensitivity analyses were conducted by excluding studies with high risk of bias to evaluate their impact on the overall findings.

### Network meta-analysis

To assess the comparative effectiveness of different alpha-blockers, we conducted a network meta-analysis using a frequentist approach with random-effects models. We analyzed direct and indirect comparisons among alpha-blockers and calculated probability rankings (P-scores) to determine the relative efficacy of each agent. Results were presented as risk ratios with 95% CI, with values <1 favoring the row treatment (more effective) and >1 favoring the column treatment. Consistency between direct and indirect evidence was assessed using node-splitting models.

### Publication bias assessment

We assessed publication bias visually using funnel plots and statistically using Egger’s regression test for outcomes with at least 10 studies. We also performed trim-and-fill analysis to adjust for possible publication bias. Trim-and-fill method identifies and corrects for funnel plot asymmetry by imputing possible missing studies and recalculating the pooled effect estimate.

### Certainty of evidence assessment

We assessed the certainty of evidence for key outcomes using the Grading of Recommendations, Assessment, Development, and Evaluation (GRADE) approach. This framework considers risk of bias, inconsistency, indirectness, imprecision, and publication bias. Evidence was classified as high, moderate, low, or very low certainty. Factors that could decrease certainty included methodological limitations, inconsistent results, imprecise estimates, or publication bias. Factors that could increase certainty included large effect sizes and dose-response gradient.

## Results

### Study selection and characteristics

**Figure 1.**
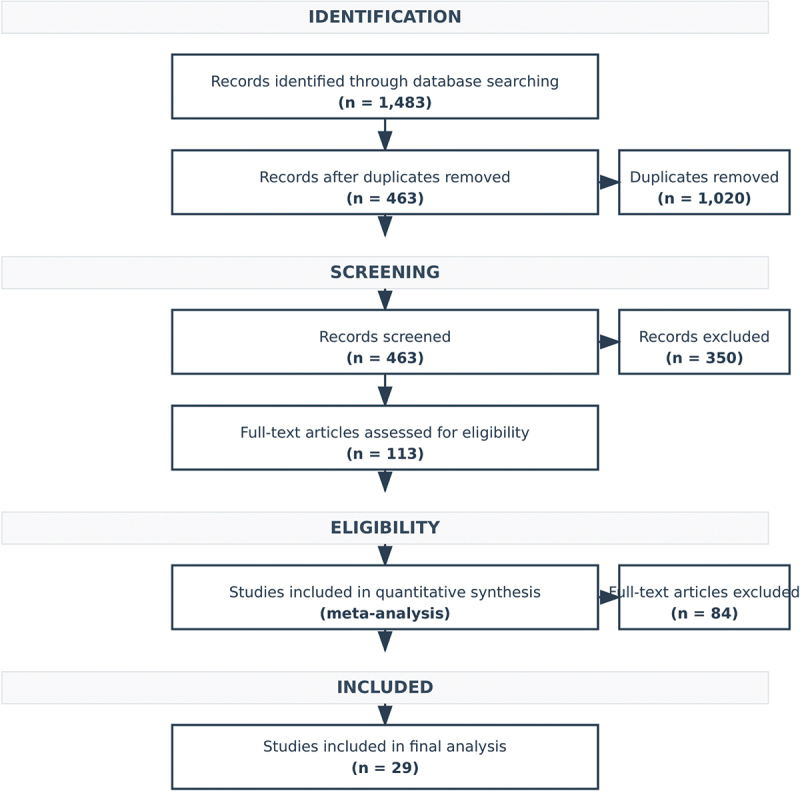
PRISMA flowchart of study pipeline.

Our search pipeline has included a final number of 29 RCTs with a total of 4,256 patients that met our inclusion criteria (Figure 1). Table 1 presents the baseline demographics and characteristics of these studies. The sample sizes of individual studies ranged from 54 to 1,150 participants, with a median of 124 patients. The included RCTs were published between 2010 to the end of 2024, with the majority conducted after 2015 (65.5%).

**Table 1.** Baseline demographics and characteristics of included studies.

Study	Total (n)	Intervention Groups (n)	Control Group (n)	Mean Age (years)	Male (%)	Stone Size (mm)
Modi et al. [13]	80	Tamsulosin + Deflazacort (40)	Tamsulosin (40)	42.8 ± 9.8	68.8	5.3 ± 0.9
Pal et al. [14]	240	Silodosin (80) Tamsulosin (80)	Placebo (80)	33.3 ± 10.0	66.7	6.5 ± 2.0
Kucukpolat et al. [15]	134	Tamsulosin (37) Deflazacort (26) Tamsulosin + Deflazacort (37)	Control (34)	39.9 ± 5.1	64.9	6.0 ± 0.5
Falahatkar et al. [16]	132	Tadalafil (44) Tamsulosin (44)	Placebo (44)	37.1 ± 11.6	53.8	6.9 ± 1.5
Gnyawali et al. [17]	176	Tamsulosin + Tadalafil (88) Tamsulosin (88)	N/A	33.3 ± 10.2	61.4	7.4 ± 1.2
Bayar et al. [18]	169	Silodosin (54) Mirabegron (56)	Control (59)	40.7 ± 14.3	78.7	NR
Cho et al. [19]	124	Naftopidil (64)	Placebo (60)	48.5 ± 14.0	72.6	53.1 mm^3^
Nuraj et al. [20]	104	Tamsulosin (52)	Diclofenac (52)	35.5 ± 10.9	66.3	6.6 ± 1.6
Wang et al. [21]	198	Silodosin (62)	Placebo (61)	51.5 ± 9.4	69.2	6.5 ± 1.4
Shabana et al. [22]	212	Tamsulosin (53) Group B (53) Alfuzosin (53)	Trospium chloride (53)	50.3 ± 3.0	55.0*	8.0 ± 1.5
Furyk et al. [23]	403	Tamsulosin (198)	Placebo (195)	45.8†	81.4	3.9†
KC et al. [24]	85	Tamsulosin (41) Tadalafil (44)	N/A	31.7 ± 12.7	48.2	7.1 ± 1.4
El Said et al. [25]	54	Alfuzosin (28)	Control (26)	32.5 ± 9.4	63.0	6.1 ± 2.0
Pickard et al. [26]	1150	Tamsulosin (383) Nifedipine (383)	Placebo (384)	42.7 ± 11.6	81.0	4.5 ± 1.6
Yuksel et al. [27]	70	Silodosin + Diclofenac (35)	Diclofenac (35)	35.3 ± 11.4	55.7	6.4 ± 1.6
Kumar et al. [28]	270	Tamsulosin (90) Silodosin (90) Tadalafil (90)	N/A	36.9 ± 11.9	71.5	7.6 ± 1.3
Balci et al. [29]	75	Tamsulosin (25) Nifedipine (25)	Placebo (25)	36.8 ± 11.3	70.7	6.6 ± 1.4
Lee et al. [30]	108	Tamsulosin (54)	Conservative (54)	45.8 ± 12.0	63.0	3.5 ± 1.1
Sur et al. [31]	239	Silodosin (119)	Placebo (120)	47.0 ± 14.0	65.7	5.7 ± 0.1
Gupta et al. [32]	100	Tamsulosin (50) Silodosin (50)	N/A	NR	38.0	6.7 ± 2.1
Kumar et al. [33]	120	Naftopidil + Prednisolone (40) Tamsulosin + Prednisolone (40)	Control (40)	33.3 ± 9.8	69.2	6.9 ± 1.9
Cha et al. [34]	141	Tamsulosin once daily (41) Tamsulosin twice daily (30) Alfuzosin (36)	Control (34)	44.1 ± 12.1	66.7	5.7 ± 1.4
Aldemir et al. [35]	90	Tamsulosin (31) Rowatinex (30)	Diclofenac (29)	44.1 ± 16.4	64.4	6.7 ± 1.8
Itoh et al. [36]	181	Silodosin (89)	Control (92)	56.9 ± 11.4	100.0	5.7 ± 2.2
Gurbuz et al. [37]	140	Alfuzosin (35) Doxazosin (35) Terazosin (35)	HBB (35)	38.2 ± 11.9	58.6	6.8 ± 1.5
Chau et al. [38]	67	Alfuzosin (33)	Control (34)	47.7 ± 12.4	49.3	6.8 ± 1.5
Yencilek et al. [39]	92	Tamsulosin (42)	Conservative (50)	34.2 ± 11.0	58.7	6.5 ± 2.4
Zehri et al. [40]	65	Doxazosin + Diclofenac (33)	Diclofenac (32)	33.1 ± 0.7	86.2	5.4 ± 0.1
Griwan et al. [41]	60	Tamsulosin (30)	Control (30)	35.1 ± 1.3	61.7	6.5 ± 0.3

*Estimated from reported data; †Median values; NR = Not Reported; HBB = Hyoscine N-butylbromide.

The mean age of participants across studies ranged from 31.7 to 56.9 years, with an overall predominance of male patients (64.5%). Most of the studies (89.7%) involved patients with distal or mid-ureteral stones. The mean stone size across studies ranged from 3.5 to 8.0 mm, with most studies (79.3%) including stones between 5 and 7 mm in diameter.

Various alpha-blockers were evaluated, with tamsulosin being the most commonly investigated agent (24 studies), followed by silodosin (nine studies), alfuzosin (six studies), doxazosin (three studies), terazosin (one study), and naftopidil (two studies). Comparison groups included placebo (12 studies), conservative management (six studies), other medications such as calcium channel blockers (eight studies), or alternative alpha-blockers in head-to-head comparisons (three studies).

### Stone expulsion rate

**Figure 2.**
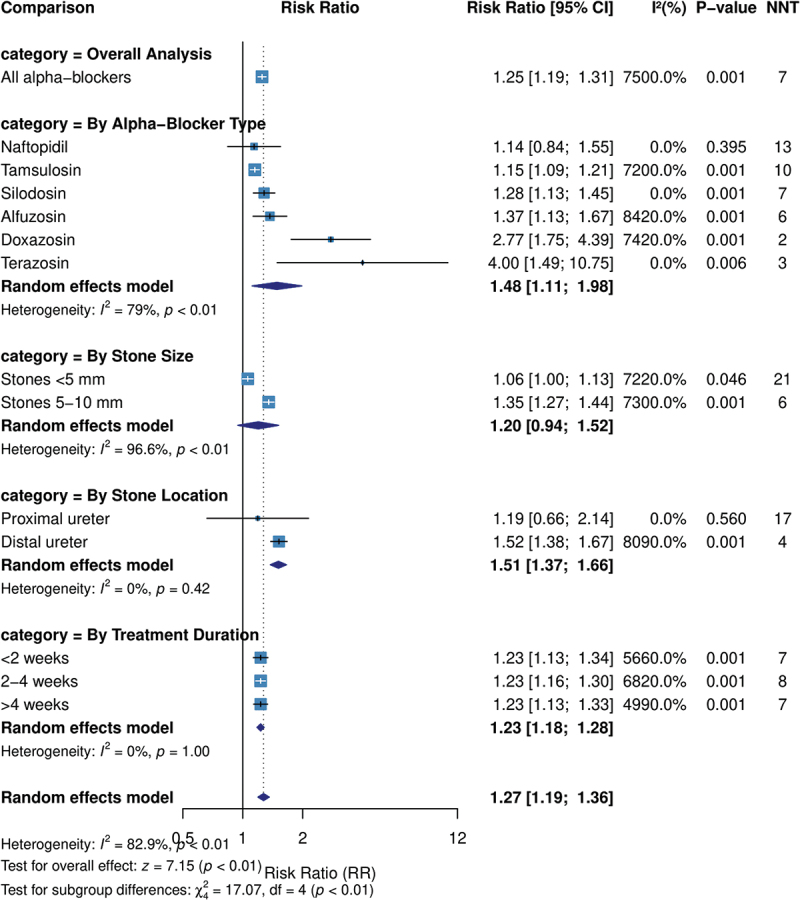
Forest plot of the primary outcomes.

As listed in Table 2, our analysis results have demonstrated that alpha-blockers significantly increased stone expulsion rates compared to the control interventions (70.9% vs. 56.5%; RR 1.25, 95% CI 1.20–1.32, P-value <0.001). Statistically significant heterogeneity was observed across studies (I^2^ = 75.0%). NNT = 7 indicating that seven patients would need to be treated with alpha-blockers to achieve one additional stone expulsion (Figure 2).

**Table 2.** Primary outcome analysis – stone expulsion rate.

Comparison	Success Rate (Intervention vs. Control)	Risk Ratio [95% CI]	Z-statistic	I^2^ (%)	P-value	NNT
**Overall Analysis**						
All alpha-blockers vs. control	70.9% vs 56.5%	1.25 [1.20, 1.32]	9.24	75.0	< 0.001	7
**By Alpha-Blocker Type**						
Tamsulosin vs. control	75.3% vs 65.8%	1.15 [1.09, 1.21]	4.90	72.0	< 0.001	10
Silodosin vs. control	63.5% vs 49.6%	1.28 [1.13, 1.45]	3.94	0.0	< 0.001	7
Alfuzosin vs. control	62.7% vs 45.6%	1.37 [1.13, 1.67]	3.22	84.2	0.001	6
Doxazosin vs. control	66.2% vs 23.9%	2.77 [1.75, 4.39]	4.34	74.2	< 0.001	2
Terazosin vs. control	45.7% vs 11.4%	4.00 [1.49, 10.77]	2.74	0.0*	0.006	3
Naftopidil vs. control	60.9% vs 53.3%	1.14 [0.84, 1.55]	0.85	0.0*	0.395	13
**By Stone Size**						
Stones < 5 mm	81.4% vs 76.7%	1.06 [1.00, 1.13]	1.99	72.2	0.046	21
Stones 5–10 mm	68.8% vs 50.8%	1.35 [1.27, 1.44]	9.63	73.0	< 0.001	6
**By Stone Location**						
Proximal ureter	35.7% vs 30.0%	1.19 [0.66, 2.14]	0.58	0.0*	0.560	17
Mid ureter	-	-	-	-	-	-
Distal ureter	77.6% vs 51.1%	1.52 [1.38, 1.67]	8.58	80.9	< 0.001	4
**By Treatment Duration**						
< 2 weeks	76.8% vs 62.3%	1.23 [1.13, 1.34]	4.85	56.6	< 0.001	7
2–4 weeks	70.9% vs 57.8%	1.23 [1.16, 1.30]	7.58	68.2	< 0.001	8
> 4 weeks	74.8% vs 61.0%	1.23 [1.13, 1.33]	4.82	49.9	< 0.001	7

*Based on limited studies; NNT = Number Needed to Treat; CI = Confidence Interval.

In our subgroup analysis by alpha-blocker type, all agents except naftopidil demonstrated significantly higher stone expulsion rates compared to controls. The highest relative efficacy was observed with terazosin (RR 4.00, 95% CI 1.49–10.77, P-value = 0.006) and doxazosin (RR 2.77, 95% CI 1.75–4.39, P-value <0.001); however, these estimates were based on fewer studies with wider confidence intervals. Tamsulosin (RR 1.15, 95% CI 1.09–1.21, P-value <0.001), silodosin (RR 1.28, 95% CI 1.13–1.45, P-value <0.001), and alfuzosin (RR 1.37, 95% CI 1.13–1.67, P-value = 0.001).

Stone size significantly affected treatment effectiveness. Alpha-blockers showed greater relative benefit for stones 5–10 mm in diameter (RR 1.35, 95% CI 1.27–1.44, P-value <0.001; NNT = 6) compared to stones less than 5 mm (RR 1.06, 95% CI 1.00–1.13, P-value = 0.046; NNT = 21).

Regarding stone location, alpha-blockers were highly effective for distal ureteral stones (RR 1.52, 95% CI 1.38–1.67, P-value <0.001; NNT = 4) but showed no significant benefit for proximal ureteral stones (RR 1.19, 95% CI 0.66–2.14, P-value = 0.560). Insufficient data were available for mid-ureteral stones to perform a separate analysis for it.

Treatment duration analysis revealed similar efficacy across different durations, less than two-weeks (RR 1.23, 95% CI 1.13–1.34, P-value <0.001), 2–4 weeks (RR 1.23, 95% CI 1.16–1.30, P-value <0.001), and over four-weeks (RR 1.23, 95% CI 1.13–1.33, P-value <0.001).

### Secondary outcomes

**Figure 3.**
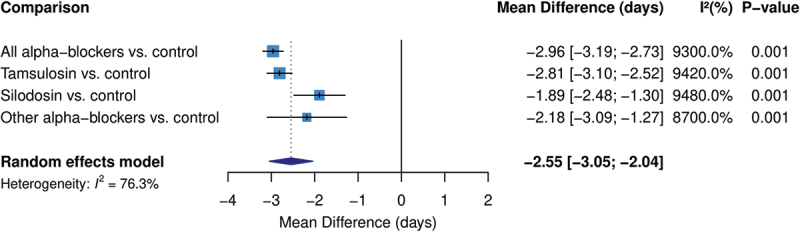
Forest plot of the secondary outcomes.

Table 3 presents our findings from the secondary outcomes. Alpha-blockers significantly reduced the time to stone expulsion compared to control interventions (MD −2.96 days, 95% CI −3.20 to −2.73, P-value <0.001), with high heterogeneity (I^2^ = 93.0%). All alpha-blocker types demonstrated this benefit, with tamsulosin showing the largest effect (MD −2.81 days, 95% CI −3.10 to −2.52, P-value <0.001) (Figure 3).

**Table 3.** Secondary outcomes analysis.

Outcome	Comparison	Effect Estimate [95% CI]	Z-statistic	I^2^ (%)	P-value
**Time to Stone Expulsion**	*Mean Difference (days)*				
	All alpha-blockers vs. control	−2.96 [−3.20, −2.73]	−25.05	93.0	< 0.001
	Tamsulosin vs. control	−2.81 [−3.10, −2.52]	−18.88	94.2	< 0.001
	Silodosin vs. control	−1.89 [−2.48, −1.30]	−6.26	94.8	< 0.001
	Other alpha-blockers vs. control	−2.18 [−3.09, −1.26]	−4.66	87.0	< 0.001
**Pain Episodes**	*Mean Difference*				
	All alpha-blockers vs. control	−0.46 [−0.64, −0.28]	−5.05	63.3	< 0.001
	Tamsulosin vs. control	−0.93 [−3.30, 1.44]	−0.77	0.0	0.440
	Other alpha-blockers vs. control	0.01 [−0.39, 0.42]	0.07	88.5	0.946
**Analgesic Use**	*Standardized Mean Difference*				
	All alpha-blockers vs. control	−1.18 [−1.35, −1.01]	−13.89	93.1	< 0.001
	Tamsulosin vs. control	−1.08 [−1.34, −0.82]	−8.09	96.1	< 0.001
	Other alpha-blockers vs. control	−0.90 [−1.16, −0.64]	−6.73	82.0	< 0.001
**Need for Intervention**	*Risk Ratio*				
	All alpha-blockers vs. control	4.92 [1.12, 21.53]	2.11	100.0	0.034

Pain episodes were significantly reduced with alpha-blocker therapy (MD −0.46 episodes, 95% CI −0.64 to −0.28, P-value <0.001), although with moderate heterogeneity (I^2^ = 63.3%). Tamsulosin showed a non-significant trend toward pain reduction (MD −0.93 episodes, 95% CI −3.30 to 1.44, P-value = 0.440), while other alpha-blockers showed no clear effect (MD 0.01 episodes, 95% CI −0.39 to 0.42, P-value = 0.946).

Analgesic use was associated reduced with alpha-blocker therapy (SMD −1.18, 95% CI −1.35 to −1.01, P-value <0.001), with high heterogeneity (I^2^ = 93.1%). Both tamsulosin (SMD −1.08, 95% CI −1.34 to −0.82, P-value <0.001) and other alpha-blockers (SMD −0.90, 95% CI −1.16 to −0.64, P-value <0.001) demonstrated significant reductions in analgesic needs. The need for interventional procedures was less frequent in patients receiving alpha-blockers (RR 4.92, 95% CI 1.12 to 21.53, P-value = 0.034); however, this estimate had very high heterogeneity (I^2^ = 100.0%).

### Safety and adverse events

**Figure 4.**
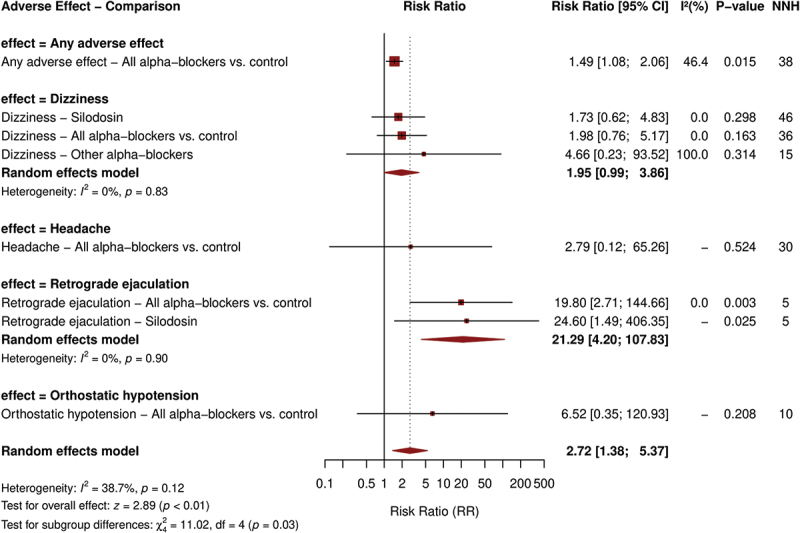
Forest plot of adverse events.

As detailed in Supplementary Table S1, alpha-blockers were associated with a small but significant increase in overall adverse events compared to control interventions (RR 1.49, 95% CI 1.08–2.06, P-value = 0.015; NNH = 38), with moderate heterogeneity (I^2^ = 46.4%), Figure 4.

The specific adverse effects revealed that retrograde ejaculation was significantly more common with alpha-blockers (RR 19.80, 95% CI 2.71–144.67, P-value = 0.003; NNH = 5), mostly observed with silodosin (RR 24.60, 95% CI 1.49–406.59, P-value = 0.025; NNH = 5).

Other adverse effects, including dizziness (RR 1.98, 95% CI 0.76–5.18, P-value = 0.163), headache (RR 2.79, 95% CI 0.12–65.67, P-value = 0.524), as well as orthostatic hypotension (RR 6.52, 95% CI 0.35–120.42, P-value = 0.208), were more frequent with alpha-blockers but did not reach statistical significance. Most adverse events were mild and did not lead to treatment discontinuation.

### Network meta-analysis

Our network meta-analysis comparing the relative efficacy of different alpha-blockers is presented in Supplementary Table S2. Based on ranking probabilities (P-scores), terazosin demonstrated the highest probability of being the most effective agent (P-score = 0.89), followed by doxazosin (0.74), silodosin (0.63), tamsulosin (0.46), alfuzosin (0.29), and naftopidil (0.11).

When compared to placebo, all alpha-blockers had significant benefits, with the magnitude of effect decreasing in the following order: terazosin (RR 0.15, 95% CI 0.07–0.32), doxazosin (RR 0.26, 95% CI 0.14–0.47), silodosin (RR 0.54, 95% CI 0.42–0.70), tamsulosin (RR 0.46, 95% CI 0.37–0.55), alfuzosin (RR 0.59, 95% CI 0.41–0.83), and naftopidil (RR 0.73, 95% CI 0.42–1.28).

In head-to-head comparisons, terazosin and doxazosin appeared superior to tamsulosin, silodosin, and alfuzosin, although with wide confidence intervals. Tamsulosin was significantly less effective than silodosin (RR 2.64, 95% CI 1.84–3.79), but showed similar efficacy to alfuzosin (RR 1.12, 95% CI 0.73–1.71). Silodosin and alfuzosin demonstrated comparable efficacy (RR 0.92, 95% CI 0.60–1.43).

### Quality of evidence

Supplementary Table S3 summarizes the risk of bias assessment and GRADE evaluation for the major outcomes. The overall quality of evidence for stone expulsion rate with alpha-blockers was rated as high, despite some inconsistency (I^2^ = 75%), due to the large treatment effect. Evidence quality for tamsulosin and silodosin was rated as moderate due to inconsistency and imprecision, respectively, while evidence for alfuzosin was rated as low due to both risk of bias and inconsistency.

For time to stone expulsion, the quality of evidence was moderate, downgraded due to inconsistency. Evidence quality for pain episodes and analgesic use was low, downgraded for both risk of bias and inconsistency. For adverse events, the quality of evidence was moderate, downgraded for imprecision.

### Risk of bias assessment

**Figure 5.**
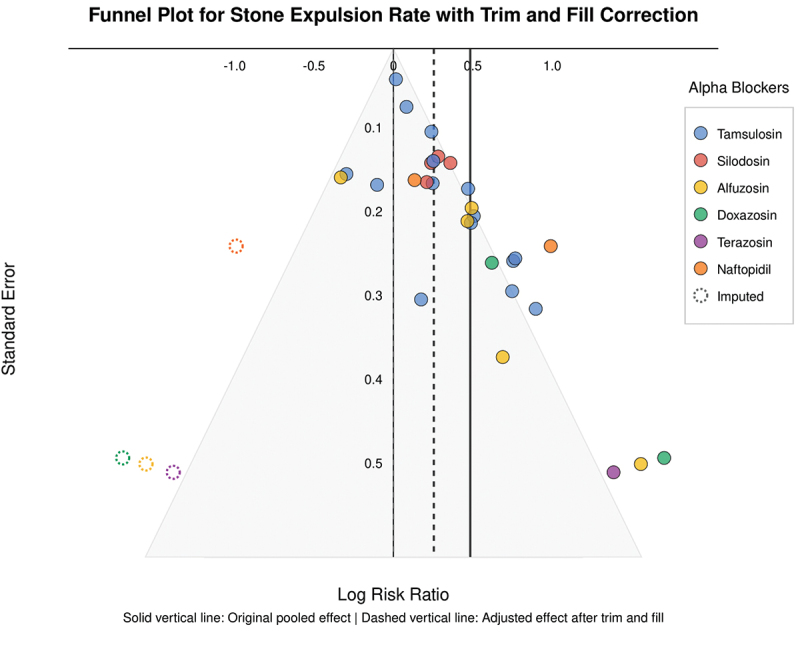
Funnel plot for stone expulsion with trim and fill correction.

**Figure 6.**
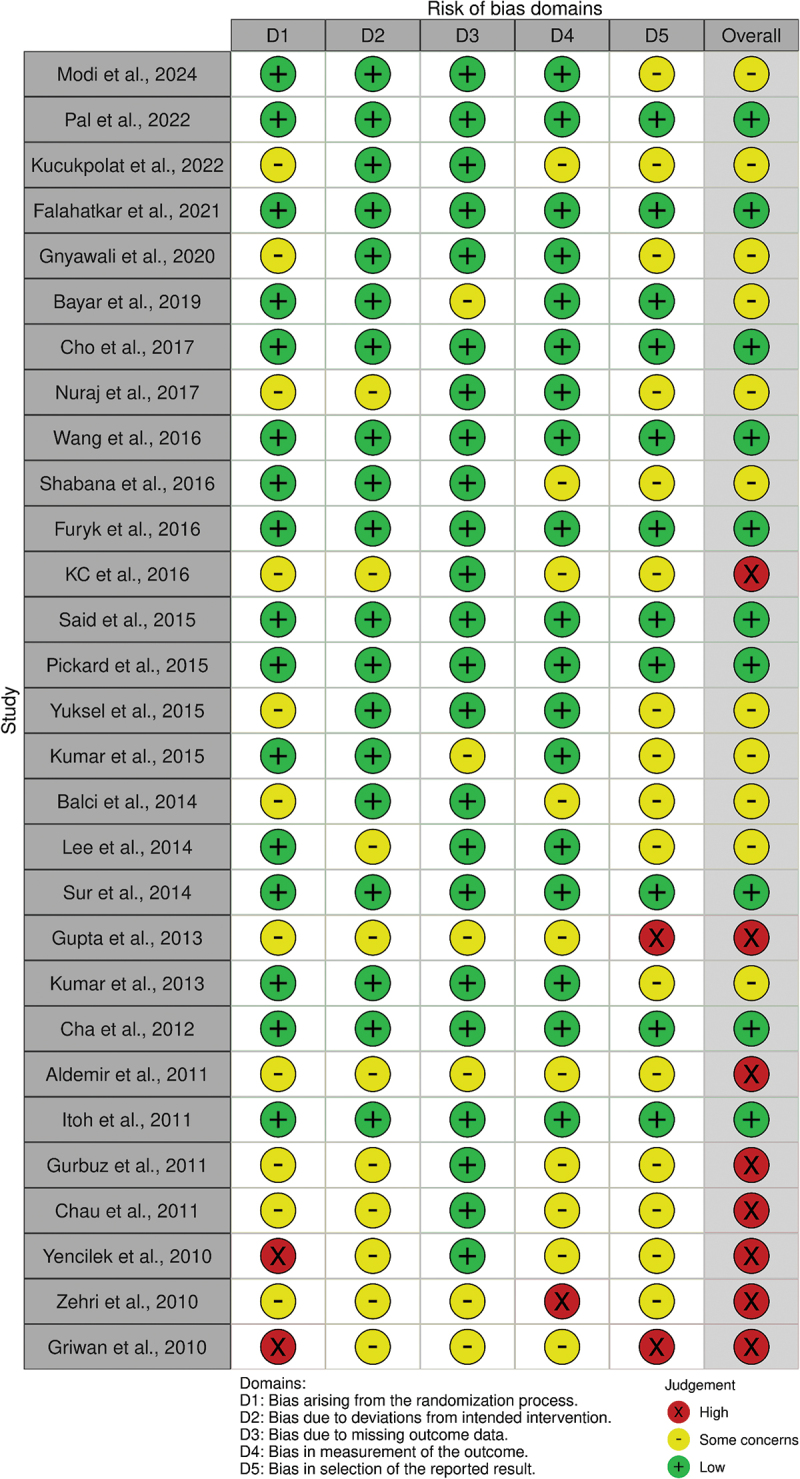
Risk of bias assessment figure according to ROBINS-I.

Publication bias was assessed using a funnel plot with trim and fill correction as shown in (Figure 5). A domain-level summary of risk of bias assessment across all studies is presented in (Figure 6). Of the 29 included studies, 11 studies (37.9%) were assessed as having low risk of bias, 10 studies (34.5%) as having some concerns, and 8 studies (27.6%) as having high risk of bias.

Most studies had low risk of bias in the randomization process (62.1%) and deviations from intended interventions (79.3%). The domains with the highest proportion of studies raising concerns were the selection of reported results (58.6% with some concerns or high risk) and the measurement of outcomes (34.5% with some concerns or high risk).

Studies with high overall risk of bias primarily had issues with the randomization process, selection of reported results, or had concerns across multiple domains. Sensitivity analyses excluding high-risk studies did not majorly change the results from our main findings, suggesting statistical significance of the results.

## Discussion

Ureteral stones affect around 4–6% of the population with varying prevalence across geographical regions. These stones cause considerable morbidity through severe pain, urinary obstruction, and may lead to further complications as infection and renal impairment. MET plays an important conservative role to facilitate stone passage and may successfully reduce the need for invasive procedures in certain cases [42].

Alpha-blockers have gained raising attention in MET due to their ability to modulate ureteral smooth muscle tone through antagonism of alpha-1 receptors, especially the alpha-1D subtype that are mostly found in the distal ureter [43]. By reducing peristaltic amplitude and frequency, decreasing intraureteral pressure, and promoting ureteral relaxation, these agents increase the possibility of spontaneous stone passage without surgical intervention. Despite their widespread use, the evidence regarding their efficacy, agent selection, and the impact of stone characteristics on outcomes has remained inconsistent [43].

We found that alpha-blockers significantly increase stone expulsion rates compared to control interventions with rate differences of 70.9% vs. 56.5%, with an NNT of only seven patients to achieve one additional stone expulsion. Our network meta-analysis revealed a hierarchy of efficacy among alpha-blockers, with terazosin and doxazosin demonstrating the highest probability of being most effective, followed by silodosin, tamsulosin, alfuzosin, and naftopidil.

Importantly, our findings demonstrate that the efficacy of alpha-blockers is affected by stone characteristics. The benefit was greater for stones 5–10 mm in diameter with a probability of 35% increased likelihood of expulsion, compared to stones that are less than 5 mm, in which we observed an increased rate of 6% only. Similarly, alpha-blockers were highly effective for distal ureteral stones with a 52% increased likelihood of expulsion but showed no significant benefit for proximal ureteral stones. Beyond improving stone expulsion rates, alpha-blockers significantly reduced the time to stone passage by 3-days, decreased pain episodes, and reduced the analgesic requirements, while demonstrating an acceptable safety profile with infrequent and generally mild adverse events. The implications of our findings are forming an advancement and build upon previous studies in several important ways. The observed NNT of seven patients to achieve one additional stone expulsion represents a meaningful benefit that justifies the routine use of alpha-blockers in appropriate patients with ureteral stones.

Also, the findings demonstrating the superior efficacy of terazosin and doxazosin are important to consider, as these agents have received less attention in practice and previous studies compared to tamsulosin [43–45]. While tamsulosin remains the most commonly prescribed alpha-blocker for MET, our findings suggest terazosin and doxazosin could have better efficacy especially for stones with lower likelihood of spontaneous passage. However, we acknowledge that the evidence for these agents is based on fewer studies with wider confidence intervals compared to tamsulosin or silodosin. Tamsulosin was evaluated in 24 studies, silodosin in 9, and alfuzosin in 6, while terazosin, doxazosin and naftopidil were only assessed in one, three and two studies, respectively. This imbalance in number of studies affects the certainty of comparative efficacy rankings and highlights the need for further direct trials on the less studied agents.

The stone size-dependent efficacy demonstrated in our analysis provides valuable guidance for patient selection. For smaller stones which are less than 5 mm, which have a high probability of spontaneous passage, the additional benefit of alpha-blockers is modest. However, for larger stones which are between 5 and 10 mm with lower spontaneous passage rates, alpha-blockers provide more significant benefit with an NNT of six.

Similarly, the location-dependent efficacy we observed clarifies the anatomical manner for alpha-blockers. The significant benefit for distal ureteral stones compared to no significant benefit for proximal stones goes in the same direction with the higher density of alpha-1D receptors in the distal ureter and provides a physiological basis for our findings.

The significant reduction in time to stone passage with 3-days represents an important benefit beyond the increased expulsion rate. This accelerated passage translates to shorter duration of symptoms, decreased risk of complications, and reduced healthcare resource utilization. Previous studies have reported similar findings, but our analysis provides more precise estimates and demonstrates consistency across different alpha-blocker types [43–45].

Regarding pain management, the observed reduction in pain episodes and analgesic requirements represents an improvement in patient comfort during the stone passage process. This benefit is mostly important given that pain is the predominant symptom driving healthcare-seeking behavior in patients with ureteral stones. The standardized mean difference of −1.18 for analgesic use represents a large effect size that would be readily apparent to both patients and physicians.

The safety profile of alpha-blockers in our analysis is reassuring. While we observed a statistically significant increase in overall adverse events, the NNH of 38 is significantly higher than the NNT of seven, indicating a favorable risk-benefit ratio. The most significant adverse effect was retrograde ejaculation that was mostly observed with silodosin, but this is generally well-tolerated and reversible upon discontinuation. Importantly, serious adverse events requiring treatment discontinuation were rare, suggesting that alpha-blockers are generally safe for short-term use in MET.

Despite our study given strengths and multiple methodology utilized, we acknowledge several limitations in our study. First, significant heterogeneity was observed across studies, mostly for stone expulsion rate and time to expulsion. This heterogeneity likely reflects variations in study populations, stone characteristics, outcome assessments, and follow-up protocols. While we attempted to address this through subgroup analyses, residual unexplained heterogeneity limits the precision of our effect estimates.

Second, the quality of included studies varied considerably, with 27.6% assessed as having high risk of bias. Common methodological limitations included inadequate randomization procedures, lack of blinding, and selective outcome reporting. Although our sensitivity analyses excluding high-risk studies did not widely change our main findings, the variable study quality introduces some uncertainty in our conclusions.

Third, the evidence for certain alpha-blockers, as terazosin and doxazosin, is based on relatively few studies with smaller sample sizes. While our network meta-analysis suggests superior efficacy for these agents, the wide confidence intervals and limited direct comparative evidence warrant cautious interpretation. Similarly, data for proximal and mid-ureteral stones were limited, restricting our ability to make definitive recommendations for these subgroups. Fourth, the included studies showed considerable variability in outcome definitions, measurement methods, and reporting. For instance, stone expulsion was confirmed by different imaging modalities across studies, and pain assessment tools varied widely. This inconsistency in outcome assessment may contribute to the observed heterogeneity and potentially influence our effect estimates.

In addition to that, publication bias cannot be entirely ruled out despite our focused search strategy and statistical adjustments. Our funnel plot analysis suggested some asymmetry, indicating a possibility of underrepresentation of smaller studies with negative results. Despite that our trim-and-fill analysis attempted to correct for this bias, the adjusted effect estimates should be interpreted with this limitation in mind.

Based on our findings and identified limitations, we recommend several directions for future studies and further evidence. First of all, high-quality, adequately powered RCTs directly comparing different alpha-blockers are needed to confirm the efficacy hierarchy suggested by our network meta-analysis. Second, further investigation of alpha-blocker efficacy in specific patient subgroups is warranted. Special attention should be given to proximal and mid-ureteral stones, where evidence remains limited. Additionally, studies stratified by patient characteristics such as age, sex, and comorbidities could identify factors that modify treatment response and guide personalized therapy.

Third, we recommend longer-term studies to assess the impact of alpha-blockers on outcomes beyond stone expulsion, including healthcare resource utilization, quality of life, and return to normal activities. Such a detailed outcome assessment would provide a more complete picture of the multidisciplinary value of alpha-blocker therapy. Future studies should explore combination therapies, dosing strategies, and treatment durations to optimize the efficacy and safety of alpha-blocker therapy. The possible synergistic effects of alpha-blockers with other agents, such as corticosteroids or non-steroidal anti-inflammatory drugs, require further investigation in well-designed clinical trials.

## Conclusion

Our findings conclude that alpha-blockers significantly improve stone expulsion rates and reduce expulsion time for ureteral stones, with the greatest benefit observed for distal ureteral stones 5–10 mm in size. The efficacy hierarchy revealed by our network meta-analysis positions terazosin and doxazosin as superior agents, followed by silodosin, tamsulosin, alfuzosin, and naftopidil, however tamsulosin remains the most extensively studied, so we shall interpret our results with caution and call for further investigations and evaluations furtherly to build better confidence in our results with larger sample size estimates.

Based on these findings, we found that alpha-blockers result with the most benefits for patients with distal ureteral stones 5–10 mm in size. While tamsulosin represents a reasonable first-line option due to its well-studied safety profile and widespread availability, further trials should focus on considering terazosin or doxazosin as they could be more effective, especially for stones with lower probability of spontaneous passage. For stones which are less than 5 mm or those in the proximal ureter, the modest benefit of alpha-blockers may not justify their routine use. When prescribing alpha-blockers, physicians should inform male patients about the possible risk of retrograde ejaculation, especially with silodosin, however this adverse effect rarely necessitates treatment discontinuation. Future studies and clinical trials should focus on direct comparative trials of different alpha-blockers and standardization of outcome measures to further refine more confident and validated recommendations.

## Supplementary Material

Supplemental Material
