# Supplementary material for: Efficacy of alpha-blockers in medical expulsive therapy for ureteral stones: A systematic review and meta-analysis of randomized controlled trials between 2010 and 2025
Source: Arab J Urol. 2025 Jul 29;24(1):1–14. doi: 10.1080/20905998.2025.2532196 (PMC12777816; doi:10.1080/20905998.2025.2532196)
Supplement: Supplemental Material [file TAJU_A_2532196_SM9937.zip › Supplementary_Table_1.docx]

**Supplementary Table 1:** Adverse Events Effect Estimates.

| **Adverse Effect** | **Comparison** | **Risk Ratio [95% CI]** | **Z-statistic** | **I² (%)** | **P-value** | **NNH** |
| --- | --- | --- | --- | --- | --- | --- |
| Any adverse effect | All alpha-blockers vs. control | 1.49 [1.08, 2.06] | 2.42 | 46.4 | 0.015 | 38 |
| **Specific Adverse Effects** | | | | | | |
| Dizziness | All alpha-blockers | 1.98 [0.76, 5.18] | 1.39 | 0.0 | 0.163 | 36 |
|  | Silodosin | 1.73 [0.62, 4.84] | 1.04 | 0.0 | 0.298 | 46 |
|  | Other alpha-blockers | 4.66 [0.23, 92.65] | 1.01 | 100.0 | 0.314 | 15 |
| Headache | All alpha-blockers | 2.79 [0.12, 65.67] | 0.64 | NaN | 0.524 | 30 |
| Retrograde ejaculation | All alpha-blockers | 19.80 [2.71, 144.67] | 2.94 | 0.0 | 0.003 | 5 |
|  | Silodosin | 24.60 [1.49, 406.59] | 2.24 | NaN | 0.025 | 5 |
| Orthostatic hypotension | All alpha-blockers | 6.52 [0.35, 120.42] | 1.26 | NaN | 0.208 | 10 |

***Notes:*** *NNH = Number Needed to Harm; CI = Confidence Interval.*
