# Supplementary material for: Efficacy of alpha-blockers in medical expulsive therapy for ureteral stones: A systematic review and meta-analysis of randomized controlled trials between 2010 and 2025
Source: Arab J Urol. 2025 Jul 29;24(1):1–14. doi: 10.1080/20905998.2025.2532196 (PMC12777816; doi:10.1080/20905998.2025.2532196)
Supplement: Supplemental Material [file TAJU_A_2532196_SM9937.zip › Supplementary_Table_2.docx]

**Supplementary Table 2:** Network Meta-Analysis and Comparative Effectiveness of Alpha-Blockers.

| **Treatment** | **Ranking Probability (P-score)** | **Compared to Placebo RR [95% CI]** | **Compared to Tamsulosin RR [95% CI]** | **Compared to Silodosin RR [95% CI]** | **Compared to Alfuzosin RR [95% CI]** |
| --- | --- | --- | --- | --- | --- |
| Terazosin | 0.89 | 0.15 [0.07, 0.32] | 0.34 [0.16, 0.72] | 0.28 [0.13, 0.61] | 0.26 [0.12, 0.59] |
| Doxazosin | 0.74 | 0.26 [0.14, 0.47] | 0.57 [0.31, 1.06] | 0.48 [0.25, 0.91] | 0.44 [0.22, 0.88] |
| Silodosin | 0.63 | 0.54 [0.42, 0.70] | 0.38 [0.26, 0.54] | — | 0.92 [0.60, 1.43] |
| Tamsulosin | 0.46 | 0.46 [0.37, 0.55] | — | 2.64 [1.84, 3.79] | 0.89 [0.58, 1.36] |
| Alfuzosin | 0.29 | 0.59 [0.41, 0.83] | 1.12 [0.73, 1.71] | 1.08 [0.70, 1.67] | — |
| Naftopidil | 0.11 | 0.73 [0.42, 1.28] | 1.61 [0.89, 2.92] | 1.35 [0.73, 2.50] | 1.25 [0.64, 2.42] |
| Placebo | 0.00 | — | 2.20 [1.81, 2.67] | 1.84 [1.43, 2.38] | 1.70 [1.20, 2.42] |

***Notes and Abbreviations:*** *RR = Risk Ratio; CI = Confidence Interval; Risk ratios <1 favor the row treatment (more effective); risk ratios >1 favor the column treatment; Treatments are ranked from most effective (top) to least effective (bottom) based on network analysis.*
