# Supplementary material for: Efficacy of alpha-blockers in medical expulsive therapy for ureteral stones: A systematic review and meta-analysis of randomized controlled trials between 2010 and 2025
Source: Arab J Urol. 2025 Jul 29;24(1):1–14. doi: 10.1080/20905998.2025.2532196 (PMC12777816; doi:10.1080/20905998.2025.2532196)
Supplement: Supplemental Material [file TAJU_A_2532196_SM9937.zip › Supplementary_Table_3.docx]

**Supplementary Table 3:** Risk of Bias and GRADE Summary of Findings.

| **Outcome** | **Comparison** | **Effect Estimate [95% CI]** | **Outcome Specific Risk of Bias Assessment** | **GRADE Certainty** | **Key Factors Affecting Certainty** |
| --- | --- | --- | --- | --- | --- |
| Stone expulsion rate | All alpha-blockers vs. control | RR 1.25 [1.20, 1.32] | Low | ⊕⊕⊕⊕ HIGH | Inconsistency (-1) Large treatment effect (+1) |
|  | Tamsulosin vs. control | RR 1.15 [1.09, 1.21] | Low | ⊕⊕⊕◯ MODERATE | Inconsistency (-1) |
|  | Silodosin vs. control | RR 1.28 [1.13, 1.45] | Low | ⊕⊕⊕◯ MODERATE | Imprecision (-1) |
|  | Alfuzosin vs. control | RR 1.37 [1.13, 1.67] | Some concerns | ⊕⊕◯◯ LOW | Risk of bias (-1) Inconsistency (-1) |
| Time to stone expulsion | All alpha-blockers vs. control | MD -2.96 [-3.20, -2.73] days | Low | ⊕⊕⊕◯ MODERATE | Inconsistency (-1) |
| Pain episodes | All alpha-blockers vs. control | MD -0.46 [-0.64, -0.28] | High | ⊕⊕◯◯ LOW | Risk of bias (-1) Inconsistency (-1) |
| Analgesic use | All alpha-blockers vs. control | SMD -1.18 [-1.35, -1.01] | High | ⊕⊕◯◯ LOW | Risk of bias (-1) Inconsistency (-1) |
| Adverse events | All alpha-blockers vs. control | RR 1.49 [1.08, 2.06] | Some concerns | ⊕⊕⊕◯ MODERATE | Imprecision (-1) |

***Abbreviations:*** *RR = Risk Ratio; MD = Mean Difference; SMD = Standardized Mean Difference; CI = Confidence Interval.*
